# Looking to the future of organ-on-chip and toxicity assessment: a regulator's opinion

**DOI:** 10.4155/fsoa-2016-0068

**Published:** 2016-10-19

**Authors:** David R Jones

**Affiliations:** 1 Medicines & Healthcare Products Regulatory Agency, 151 Buckingham Palace Road, London, SW1W 9SZ, UK

**Keywords:** organ-on-chip, pharmacology, regulation, toxicology

## Abstract

**David R Jones talks to Francesca Lake, Managing Editor:** David R Jones is an Expert Pharmaco-Toxicologist within the Licensing Division of the Medicines and Healthcare products Regulatory Agency. We met him at the Organ-on-a-chip Europe 2016 conference in Cambridge (UK), where he presented ‘A UK Regulatory View on the Acceptability of Organ on a Chip Data’.

## Q Can you tell us a little about your career to-date, & what your current roles entail?

I spent 8 years working in a contract research organization and 11 years as a toxicologist within the pharmaceutical industry. I joined the Medicines Control Agency in 1996, which later changed its name to the Medicines and Healthcare Products Regulatory Agency (MHRA) following a merger with the Medical Device Agency in 2003. I currently work as an Expert Pharmaco-Toxicologist within the Licensing Division and my role principally involves assessing nonclinical data for clinical trial authorization applications. I also take part in meetings offering scientific and regulatory advice to companies and academia on behalf of the MHRA or the EU's Committee for Human Medicinal Products (CHMP). I am one of the UK's accredited nonclinical experts to support the CHMP and am also the UK representative on the safety working party. I represent the safety working party within the International Council for Harmonization and am currently working on the development of the new International Council for Harmonization S11 guideline on Nonclinical Safety Testing in Support of Development of Pediatric Medicines.

I also work closely with the UK's National Centre for the Replacement, Refinement and Reduction of animals in research (NC3Rs) and spend a lot of my time in supporting groups developing alternatives to *in vivo* animal testing.

Finally, I am also a guest lecturer at the University of Surrey and the University of Wales and a frequent presenter at conferences around the world.

## Q What does a typical day look like for you?

I am in a fortunate situation where there really is never a ‘typical’ day. However, my primary duty is the safety evaluation of clinical trial authorization applications for new medicines, which involves reviewing all the nonclinical data generated from primary pharmacology through to carcinogenicity studies.

## Q What do you find most challenging in your work?

The most challenging part of my work is the safety assessment of ‘first in human’ clinical trials. In these cases, the nonclinical data are the only safety information available and it is essential to set a safe starting dose, ensure inclusion and exclusion criteria are acceptable, identify things to be monitored in the clinical trial and to identify a safe maximum dose to be evaluated. If the trial is in patients rather than healthy volunteers, then there is an additional pressure to ensure that while the starting dose is safe, it still has the potential of offering a degree of therapeutic benefit.

## Q You work closely with the NC3Rs & represent the MHRA on a governmental body dealing with animal welfare in research: what are their thoughts on organ-on-a-chip-use in drug development?

I really enjoy working with the NC3Rs. They collaborate with scientists and organizations from across the life sciences sector, nationally and internationally, including universities, the pharmaceutical, chemical and consumer products industries, other research funders and regulatory authorities. They also fund 3Rs’ research, training and career development, support open innovation and commercialization of 3R technologies, and stimulate changes in policy, regulations and practice relating to the use of animals. The NC3Rs have long supported organ-on-chip technology and, indeed, their 2012 3Rs Prize winner was Professor Donald Ingber at the Wyss Institute for Biologically Inspired Engineering, Harvard University, USA, for a lung-on-a-chip microdevice [1].

## Q Besides organ-on-a-chip, what other promising research is currently happening to improve or reduce our use of animals in research?

Across the biosciences there are a great many techniques which do not involve animals. Even within projects involving animal research there may be large amounts of work carried out in human tissue samples. Scientists are constantly working on ways to reduce the use of animals in research and the MHRA's work in this area was outlined in a 2014 government publication “Working to reduce the use of animals in scientific research” [2]. A paper entitled ‘Waiving *in vivo* studies for monoclonal antibody biosimilar development: national and global challenges’ was also published in the journal *mAbs* recently and the authors include myself, other MHRA Toxicologists and scientists from the NC3Rs [3].

## Q That paper recommends a global program is needed to improve the efficiency of biosimilar development & make a move toward more *in vitro* testing. What challenges do you face in making these recommendations a reality, & how do you foresee us overcoming them?

Pharmaceutical development is already a global program for new drugs and to expand that to the biosimilar arena should not be too challenging. There are already international guidelines for biosimilar development and they are not too dissimilar. The MHRA has a program on nonclinical support for biosimilar development running in conjunction with the NC3Rs and through them we are already challenging companies to adopt a more scientific approach. The NC3Rs has published a paper on this issue too.

## Q Organ-on-a-chip research seems to be quite far from meeting public expectancy that it will replace animal use. Do you think it ever will, & why? Could public hype damage its development?

I personally think that organ-on-chip technology will become an integral part of the safety evaluation of new medicines, but I am concerned that public hype currently does exceed what is technically feasible. This was also mentioned in an article in *Nature* by Sara Reardon [4]. One additional role I have at the MHRA is writing responses to letters from the public an animal testing on new medicines and we are frequently being asked why organ-on-chip models have not replaced studies in animals yet. I do worry that companies could abandon work in this area if the technology fails to live up to people's expectations.

## Q What barriers do you think are preventing the wider uptake of tissue-based research in early drug development?

There are a couple of genuine barriers and some perceived ones too. There always appears to be a perception that regulators like myself are not interested in new technologies unless they are fully validated. This is simply not true and regulators are keen to engage in areas of animal alternatives. Many regulators, including myself, are involved in projects through the Innovative Medicines Initiative [5] or the ILSI Health and Environmental Sciences Institute [6].

The ‘real barriers’ will always be validation, not least because most people cannot agree what validation actually is or how to go about it. Here again, regulators do try to help and the EU is writing a guideline in this area – Guideline on regulatory acceptance of 3R (replacement, reduction, refinement) testing approaches (EMA/CHMP/CVMP/JEG-3Rs/450091/2012).

## Q Ideally, how would you like toxicity assessment to look in 20 years’ time?

I have seen toxicology assessment evolving over the last 30 years and I think that will continue. I look forward to the assessment becoming increasingly more science-based rather than focusing on regulatory guidelines. I certainly think we will see more human cell based assays and organ-on-chip technology being used and an improvement in *in silico* methods. I think we will see far more *in vitro* and *in silico* methods being used early in development, but think we will still be using some animals unless there have been some major breakthroughs in our understanding in the real underlying causes of human diseases.
